# Pulmonary vasodilator therapy for patients after Fontan procedure: A protocol for a systematic review and meta-analysis

**DOI:** 10.1097/MD.0000000000017615

**Published:** 2019-10-18

**Authors:** Dongxu Li, Xu Zhou, Qi An, Yuan Feng

**Affiliations:** a Department of Cardiovascular Surgery, West China Hospital, Sichuan University, Chengdu; b Evidence-based Medicine Research Center, School of Basic Medical Sciences, Jiangxi University of Traditional Chinese Medicine, Jiangxi; c Department of Cardiology, West China Hospital, Sichuan University, Sichuan, PR China.

**Keywords:** exercise capacity, Fontan procedure, heart function, pulmonary resistance, pulmonary vasodilator, safety

## Abstract

**Background:**

The Fontan circulation is fragile, which is easily broken down. For now, there is no consensus on the drug treatment for the prevention of failure of the Fontan circulation.

**Methods:**

Studies comparing pulmonary vasodilator and control in Fontan patients were identified by searching the PubMed, EMBASE, Clinical Trials, and the Cochrane Library databases until March 20, 2019. The assessed variables included the change of pulmonary resistance, heart function, exercise capacity, life of quality, mortality, and serials of adverse events before and after drug administration. A random-effect/fixed-effect model was used to summarize the estimates of the mean difference (MD)/risk ratio (RR) with 95% confidence interval (CI). Subgroup analysis stratified by drug was performed.

**Results:**

This study will be submitted to a peer-reviewed journal for publication.

**Conclusion:**

This study will assess the efficacy and safety of pulmonary vasodilators for patients after Fontan procedure, and provide more evidence-based guidance in clinical practice.

**PROSPERO registration number:**

CRD42019132135.

## 1 Introduction

The Fontan procedure has provided excellent surgical palliation through total cavopulmonary connection for patients with single ventricle.^[1]^ Since its introduction in 1971, several modifications have resulted in decrease in incidence of postoperative complications and death.^[2–6]^ However, survivors of Fontan palliation mostly have diminished exercise tolerance and easily have a failure of Fontan-pulmonary circulation.^[7]^ One of the most important reasons is that the absence of pre-pulmonary ventricle fails to pump blood through vasculature to maintain the necessary cardiac output which also can be affected by the change of pulmonary vascular resistance (PVR).^[8]^

It has been suggested that reduced PVR and pulmonary arterial pressure (PAP) could result in increased pulmonary blood flow and better cardiac filling which thereby allows an improved cardiac output response to exercise.^[9]^ Consequently, the pulmonary vasodilator has been identified as a potential therapy for patients with Fontan physiology.^[10]^ Pulmonary vasodilators involving in post-Fontan treatment mainly include the phosphodiesterase-5 inhibitors such as sildenafil and tadalafil, the endothelin receptor antagonists such as bosentan and ambrisentan, and the prostacyclins such as iloprost and beraprost.^[11]^ Although these drugs have been reported to be administrated in clinical trials, there is no consensus on the optimal drug treatment for the prevention of failure of the Fontan circulation.^[10]^

Therefore, we aimed to perform this meta-analysis to assess the efficacy and safety of such pulmonary vasodilators for patients after Fontan palliation, and hope to support evidence for clinical strategy.

## 2 Methods

### 2.1 Study registration

This protocol is conducted according to the Preferred Reporting Items for Systematic Review and Meta-Analysis Protocols (PRISMA-P) statement.^[11]^ We will report the results of this systematic review and meta-analysis adhere to the Preferred Reporting Items for Systematic Reviews and Meta-Analysis (PRISMA) guidelines.^[12]^ This protocol has been registered in the PROSPERO network (registration number: CRD42019132135).

### 2.2 Eligibility criteria

#### 2.2.1 Types of studies

Two-arm studies including a randomized controlled trial (RCT), a prospective or retrospective cohort with comparison of objective drug and control will be incorporated in this study without published year, publication status limitations.

#### 2.2.2 Types of participants

The patients with congenital cardiac anomalies underwent Fontan procedure will be included. There will be no restrictions on sex, ethnicity, economic status, and education.

#### 2.2.3 Types of interventions and comparators

The treatment group will be treated with pulmonary vasodilators such as phosphodiesterase-5 inhibitor, endothelin receptor antagonist, or prostacyclin. The control group will be treated with placebo or nothing.

#### 2.2.4 Types of outcome measures

The primary outcomes are the changes of pulmonary resistance, heart function, exercise capacity, and mortality after drug administration. The secondary outcomes are the changes of quality of life and adverse events.

### 2.3 Search strategy

A literature search of computerized medical literature was performed using the PubMed, EMBASE, Clinical Trials, and the Cochrane Library databases. The keywords searched were “Fontan” and “pulmonary vasodilator”. The search was conducted for published papers from the inception of the databases until March 20, 2019, and there were no language restrictions. To ensure that the search was complete, the reference lists of all retrieved articles were manually searched to identify additional relevant studies by 2 reviewers (DXL, XZ).

### 2.4 Data collection and analysis

#### 2.4.1 Data management

The search results from above 4 databases will be imported to Endnote X7 software (Thomson Reuters, Canada) to data management. Before the literature selection, the study criteria will be conducted between the reviewers to ensure high inter-rater agreement.

#### 2.4.2 Study selection

Two reviewers (DXL, XZ) will independently evaluate the title and abstract of all studies for possible candidates. Any duplicate studies will be removed. After passing the title and abstract screening, the full-text copies of all eligible studies will be downloaded for re-evaluation. If the reviewer is uncertain about the eligibility of any study, its full text will be obtained to re-examine. An additional reviewer (QA or YF) will be consulted in case of disagreement. Excluded studies and the reasons of exclusion will be recorded. We will show the selection process in details in the PRISMA flow chart.^[11]^

#### 2.4.3 Data collection process

Two independent researchers extracted data (DXL, XZ). Any disagreements will be resolved by a third reviewer (YF). The following data were extracted from each eligible study using a standardized data collection form: first author's name, study design, publication year, country where the study was conducted, sample size, age, weight, gender, main diagnosis, Fontan type, fenestration, drug dosage, and follow-up interval. Primary outcomes such as pulmonary resistance involving PVR or PAP, heart function involving New York Heart Association (NYHA) class or brain natriuretic peptide (BNP) or pro-BNP, exercise capacity involving postoperative arterial oxygen saturation (SaO2) at rest, or peak oxygen consumption (VO2), and postoperative mortality will be collected. Additional outcomes such as quality of life involving Short Form-36, and any adverse event involving protein-losing enteropathy, headache, flushing, hepatic dysfunction, edema, nausea, cough/dyspnea, fatigue, diarrhea, cutaneous discomfort (itching or rash), and somatic pain will be collected.

### 2.5 Quality of evidence assessment

According to Grading of Recommendations Assessment Development and Evaluation (GRADE), the quality of included studies will be assessed by the online guideline development tool http://gdt.guidelinedevelopment.org/), and divided into 4 levels: high quality, moderate quality, low quality, and very low quality.^[13]^

### 2.6 Assessment of risk of bias in included studies

The risk of bias was assessed independently by 2 reviewers using the Cochrane Collaboration tool.^[14]^ Every domain was scored as high risk of bias, low risk of bias, or unclear. The overall assessment of each study was graded as“low risk” (if all domains were assessed as low risk of bias), “unclear” (if at least 1 domain was assessed as unclear), or “high risk” (otherwise).

### 2.7 Data analysis

The measures of the effects of interest were the risk ratio (RR)/mean difference (MD) with 95% confidence intervals (CIs), in which the MDs were combined using the inverse variance method.^[15]^ We imputed the missing standard deviation (SD) of the change between before and after drug administration using the following formula recommended in the Cochrane Handbook: 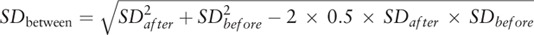.^[15]^ We used the Cochrane Chi-Square test (*Q* test) and the *I*^2^ test to evaluate level of heterogeneity across studies. When significant heterogeneity (*P* < . 05 or *I*^2^ > 50%) was detected, we pooled data using a random-effect model.^[16]^ Otherwise, a fixed-effect model was used.^[17]^ We explored the source of heterogeneity using sensitivity analysis. Funnel plots were visually inspected to identify any potential publication bias. All statistical analyses were performed using Review Manager software (version 5. 3; Cochrane Collaboration, Oxford, UK) and Stata software (version 14. 0; Stata Corp., College Station, TX, USA).

#### 2.7.1 Subgroup analysis

If significant heterogeneity is detected between studies, we will explore whether the results are inconsistent in different subgroups. Subgroup analysis will be performed stratified by drug category.

#### 2.7.2 Sensitivity analysis

Sensitivity analysis will be conducted to assess the reliability and robustness of the aggregation results via eliminating trials with high bias risk.

#### 2.7.3 Publication bias

According to Cochrane Handbook, if there are ≥10 trials included, we will construct a funnel plot and use Egger test to assess publication bias.^[15]^

### 2.8 Ethics and dissemination

The study does not require ethical approval because the original data are anonymous, which no privacy will be involved. And this study will eventually be published in a peer-reviewed journal in the form of a scientific paper.

## Author contributions

**Data curation:** Dongxu Li, Xu Zhou.

**Formal analysis:** Dongxu Li, Xu Zhou.

**Methodology:** Dongxu Li, Xu Zhou.

**Project administration:** Dongxu Li.

**Supervision:** Yuan Feng.

**Writing – original draft:** Dongxu Li.

**Writing – review & editing:** Dongxu Li, Xu Zhou, Qi An, Yuan Feng.

## References

[1] Li D Fan Q Hirata Y Arrhythmias after fontan operation with intra-atrial lateral tunnel versus extra-cardiac conduit: A Systematic Review and Meta-analysis. Pediatr Cardiol 2017;38:873 80. 28271152 10.1007/s00246-017-1595-8

[2] Fontan F Baudet E Surgical repair of tricuspid atresia. Thorax 1971;26:240 8. 5089489 10.1136/thx.26.3.240 PMC1019078

[3] Puga FJ Chiavarelli M Hagler DJ Modifications of the Fontan operation applicable to patients with left atrioventricular valve atresia or single atrioventricular valve. Circulation 1987;76 (3 Pt 2):III53 60. 2441897

[4] de Leval MR Kilner P Gewillig M Total cavopulmonary connection: a logical alternative to atriopulmonary connection for complex Fontan operations. Experimental studies and early clinical experience. J Thorac Cardiovasc Surg 1988;96:682 95. 3184963

[5] Bridges ND Mayer JE Jr Lock JE Effect of baffle fenestration on outcome of the modified Fontan operation. Circulation 1992;86:1762 9. 1451248 10.1161/01.cir.86.6.1762

[6] Jonas RA The intra/extracardiac conduit fenestrated fontan. Semin Thorac Cardiovasc Surg Pediatr Card Surg Annu 2011;14:11 8. 21444043 10.1053/j.pcsu.2011.01.010

[7] Stephen M Paridon Paul D A cross-sectional study of exercise performance during the first two decades of life following the Fontan operation. J Am Coll Cardiol 2008;52:99 107. 18598887 10.1016/j.jacc.2008.02.081

[8] Gewillig M Brown SC Eyskens B The Fontan circulation: who controls cardiac output? Interact Cardiovasc Thorac Surg 2010;10:428 33. 19995891 10.1510/icvts.2009.218594

[9] La Gerche A Gewillig M What limits cardiac performance during exercise in normal subjects and in healthy Fontan patients? Int J Pediatr 2010;791291. 20871839 10.1155/2010/791291 PMC2943078

[10] Oldenburger NJ Mank A Etnel J Drug therapy in the prevention of failure of the Fontan circulation: a systematic review. Cardiol Young 2016;26:842 50. 26947621 10.1017/S1047951115002747

[11] Shamseer L Moher D Clarke M Preferred reporting items for systematic review and meta-analysis protocols (PRISMA-P) 2015: elaboration & explanation. BMJ 2015;349:g7647. 10.1136/bmj.g7647 25555855

[12] Moher D Liberati A Tetzlaff J Preferred reporting items for systematic reviews and meta-analyses: The PRISMA statement. Int J Surg 2010;8:336 41. 20171303 10.1016/j.ijsu.2010.02.007

[13] Puhan MA Schünemann HJ Murad MH A GRADE Working Group approach for rating the quality of treatment effect estimates from network meta-analysis. BMJ 2014;349:g5630. 25252733 10.1136/bmj.g5630

[14] Higgins JP Altman DG Gotzsche PC The Cochrane Collaboration's tool for assessing risk of bias in randomised trials. BMJ 2011;343:d5928. 22008217 10.1136/bmj.d5928 PMC3196245

[15] The Cochrane Collaboration, Higgins JP Green S Cochrane Handbook for Systematic Reviews of Interventions (Version 5. 1). 2011;http://handbook-5-1.cochrane.org/http://handbook-5-1.cochrane.org/ [accessed March 20, 2019].

[16] DerSimonian R Kacker R Random-effects model for meta-analysis of clinical trials: an update. Contemp Clin Trials 2007;28:105 14. 16807131 10.1016/j.cct.2006.04.004

[17] Higgins JP Thompson SG Deeks JJ Measuring inconsistency in meta-analyses. BMJ 2003;327:557 60. 12958120 10.1136/bmj.327.7414.557 PMC192859

